# Adaptation to a Whole-Body Powered Exoskeleton: Human–Exoskeleton Coordination During Load-Handling Tasks

**DOI:** 10.1007/s10439-026-04025-9

**Published:** 2026-03-10

**Authors:** Hanjun Park, Sunwook Kim, Maury A. Nussbaum, Divya Srinivasan

**Affiliations:** 1 Department of Industrial, Manufacturing, and Systems Engineering, Texas Tech University, Lubbock, TX 79409 USA; 2 Department of Industrial and Systems Engineering, Virginia Tech, Blacksburg, VA 24061 USA; 3 Department of Industrial Engineering, Clemson University, Clemson, SC 29634 USA

**Keywords:** Motor adaptation, Learning, Electromyography, Kinematics, Cognitive workload

## Abstract

Whole-body powered exoskeletons can augment human performance and reduce physical strain in occupational settings, but little is known about how users adapt to these complex devices during practical work scenarios. We compared novice and experienced users during simulated, occupationally relevant load-handling tasks. Six novice users completed exoskeleton familiarization and stationary load-handling tasks in three sessions while five experienced users performed the tasks once. Task performance, biomechanical demands, and perceived workload were compared in each novice session vs. the experienced group. Novice performance improved substantially across sessions, with task completion time reduced by nearly 50% and movement jerk by 30%. However, performance gaps still persisted in session three, compared to the experienced users. Novices also used consistently lower angular velocities (up to 52% lower) and adopted greater hip flexion throughout the sessions. In contrast, differences in shoulder flexion, muscle activity, perceived exertion, and workload diminished more rapidly, with novices approaching experienced levels by session three. Novice users adapted to using a powered exoskeleton over multiple sessions, especially in movement patterns and muscle activation, but differences in task completion time, jerk index, and angular velocities indicated that novices did not attain the skilled coordination and efficiency of experienced users after three sessions. Our results highlight the likely need for extended familiarization and training for the current powered exoskeleton design and provide baseline data for the novice learning curve in occupational settings.

**Supplementary Information:**

The online version contains supplementary material available at https://doi.org/10.1007/s10439-026-04025-9.

## Introduction

Exoskeletons are increasingly used in occupational settings to reduce physical demands and to mitigate risk factors associated with work-related musculoskeletal disorders (WMSDs). Exoskeletons can reduce physical effort (measured both objectively and subjectively), such as during lifting (e.g., [1, 2]), carrying (e.g., [3, 4]), and construction tasks (e.g., [5, 6]), although evidence of long-term WMSD prevention is still emerging. Commonly, exoskeletons are categorized as passive or active/powered based on their actuation mechanisms (e.g., [7, 8]). Passive devices use non-powered elements such as springs or elastic materials, while powered devices incorporate actuators and control systems to align assistance with user intent. Powered exoskeletons are often presumed to offer greater potential for augmentation and versatility [8], and several studies have reported greater reductions in oxygen consumption and muscle activity with powered compared to passive devices during manual handling tasks [9–11]. However, other studies have found increased workload with both powered and passive devices during industrial tasks [12] and elevated cognitive workload during construction tasks [13]. These inconsistent findings may be due to differences in assistive torques provided by the devices, as well as complexities in being able to leverage device design and capability to specific occupational tasks.

Currently, most commercially available exoskeletons target a single body region, primarily supporting either the back/hips or the arms. Back-support exoskeletons have been extensively studied for trunk bending and lifting [14, 15] and arm-support exoskeletons for overhead or arm-elevation work (e.g., [16–18]). Both exoskeleton types have demonstrated effectiveness in reducing physical demands, reflected in decreased muscle activity, subjective strain, and energy expenditure. However, it is largely unknown whether such findings generalize to more complex, multi-joint powered exoskeletons, particularly given limited evidence regarding the training and motor adaptation required for users of these more sophisticated systems.

Motor adaptation to exoskeletons likely varies depending on the number of actuated joints and the level of assistance provided. Kao et al. [19] reported longer adaptation periods for lower-limb dynamics during walking when using exoskeletons offering greater torque assistance. As such, individuals could take longer to adapt to an exoskeleton that provides greater assistance, though the precise relationship between the adaptation time scale and the complexity or degree of provided assistance remains unclear. Given differences in neuromuscular coordination across joints [20, 21], adaptation to multi-joint exoskeletons, with their greater complexity and intricate control systems, can be expected to be more demanding and prolonged for users [22].

We previously reported three investigations of a whole-body powered exoskeleton, the ‘pre-alpha Sarcos prototype’. This device included 18 actuated degrees of freedom spanning multiple joints and providing substantially greater mechanical assistance compared to typical single-joint devices. In our first study, we identified appropriate load levels for effective use of the device in load-handling tasks [23], finding benefits only for loads exceeding ~10 kg (i.e., reduced activity of the back and leg muscles). In a second study, we examined gait among experienced users and found altered movement strategies, such as greater reliance on hip motions and time-lagged muscle activity when using the device relative to unassisted walking [24]. In a third study, we examined novice adaptation during gait and found that novice movement patterns did not match those of experienced users even after three sessions [25].

In this study, we have expanded on this earlier work by investigating the adaptation of novices when using a whole-body powered exoskeleton during simulated load-handling tasks. Our goal was to characterize how human–exoskeleton coordination develops during a physically demanding occupational task, in terms of task performance, movement smoothness, joint kinematics, muscle activity, and perceived workload. This work is novel in two key respects. First, unlike prior exoskeleton studies that focused primarily on gait or isolated upper-limb assistance, the present study examined whole-body coordination during load-handling, a task that emphasizes upper–lower body coupling, force transmission through the trunk, and coordination between the user, the device, and an external object. Second, load-handling is a coordination challenge that differs fundamentally from locomotion, so adaptation trajectories observed during gait cannot be assumed to generalize to occupational lifting tasks. We use the term *adaptation* to refer to experience-dependent changes in how tasks are performed with the exoskeleton, and *coordination* to refer to the quality and consistency of movement patterns used to interact effectively with the device. We hypothesized that novices would adapt more rapidly to load-handling than to dynamic gait tasks, with both objective and subjective measures converging toward values observed in experienced users within three sessions. We further hypothesized that performance–quality measures indicating coordination efficiency (e.g., task completion time and movement smoothness) would exhibit slower improvement, since these measures are more closely tied to skill acquisition. Prior motor learning research has shown that such measures improve gradually as learners shift from feedback-dominated corrections to more efficient, feed-forward control supported by internal model formation [26, 27].

## Methods

### Participants

**Fig. 1:**
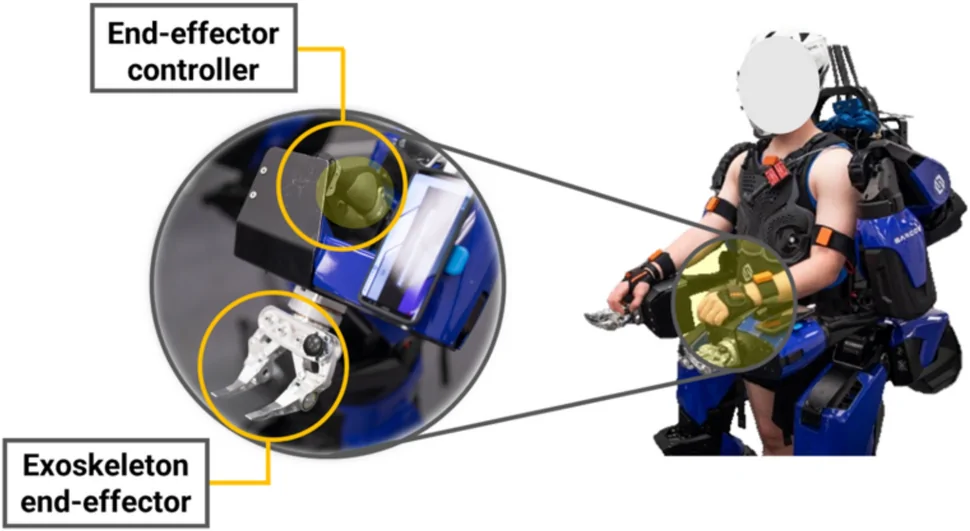
Pre-alpha prototype of the occupational whole-body powered exoskeleton tested (Guardian® XO®, Sarcos Robotics, https://www.sarcos.com [29]). Yellow circled areas denote the exoskeleton end-effector and a controller, which is embedded with a force-moment sensor that allows the exoskeleton to measure human input forces. Yellow shaded areas denote load cell interfaces embedded within the controller

Eleven healthy male participants (6 novices and 5 experienced users) completed the study, all of whom reported no musculoskeletal injuries or disorders in the prior year and met the exoskeleton’s anthropometric constraints. Regarding the latter, the whole-body powered exoskeleton prototype was configured for individuals 1.82 (± 0.03) m tall to ensure proper contact with load cell interfaces located at hands, feet, torso, and pelvis (Fig. 1). Novices were defined as those who had no prior experience operating the whole-body powered exoskeleton. Experienced participants had ≥ 3 months of prior experience in use during the prototype development phase, and their proficiency was confirmed by investigators and manufacturer engineers based on demonstrated operational skills. Prior to any data collection, informed consent was obtained from each participant, and the study procedures were approved by the Virginia Tech Institutional Review Board.

### Whole-Body Powered Exoskeleton

A pre-alpha prototype of the Guardian® XO®, developed by Sarcos Robotics, was used in the current study (Fig. 1). The XO® has a mass of 158 kg and includes 18 active degrees-of-freedom spanning the shoulders, elbows, trunk, hips, and knees. The system provides lift assistance, allowing users to handle loads up to 90 kg. While full details of the control algorithms and specific parameter values are proprietary and therefore cannot be disclosed, the general control framework implemented in the whole-body powered exoskeleton has been described in a prior peer-reviewed publication and patent [25, 28]. Briefly, the exoskeleton uses torque sensing at major body joints to follow the user’s movements and to amplify human joint torques during load-handling tasks. User movement intent is inferred from embedded six-degree-of-freedom load cells located at the hands, feet, torso, and pelvis, enabling coordinated whole-body assistance during lifting and carrying. The system includes several tunable control parameters, such as virtual center-of-mass settings, gravity compensation, and actuation gains that determine the magnitude of torque amplification. In the present study, these parameters were adjusted during an initial familiarization session to ensure safe and comfortable operation for each participant. Once configured, all control parameters were held constant for a given participant across all experimental sessions and trials. No adaptive, session-dependent, or trial-to-trial modifications to control gains or thresholds were implemented during data collection. Accordingly, any observed changes in performance are driven by human adaptation to the device rather than changes in exoskeleton control behavior.

### Experimental Design and Procedures

**Fig. 2:**
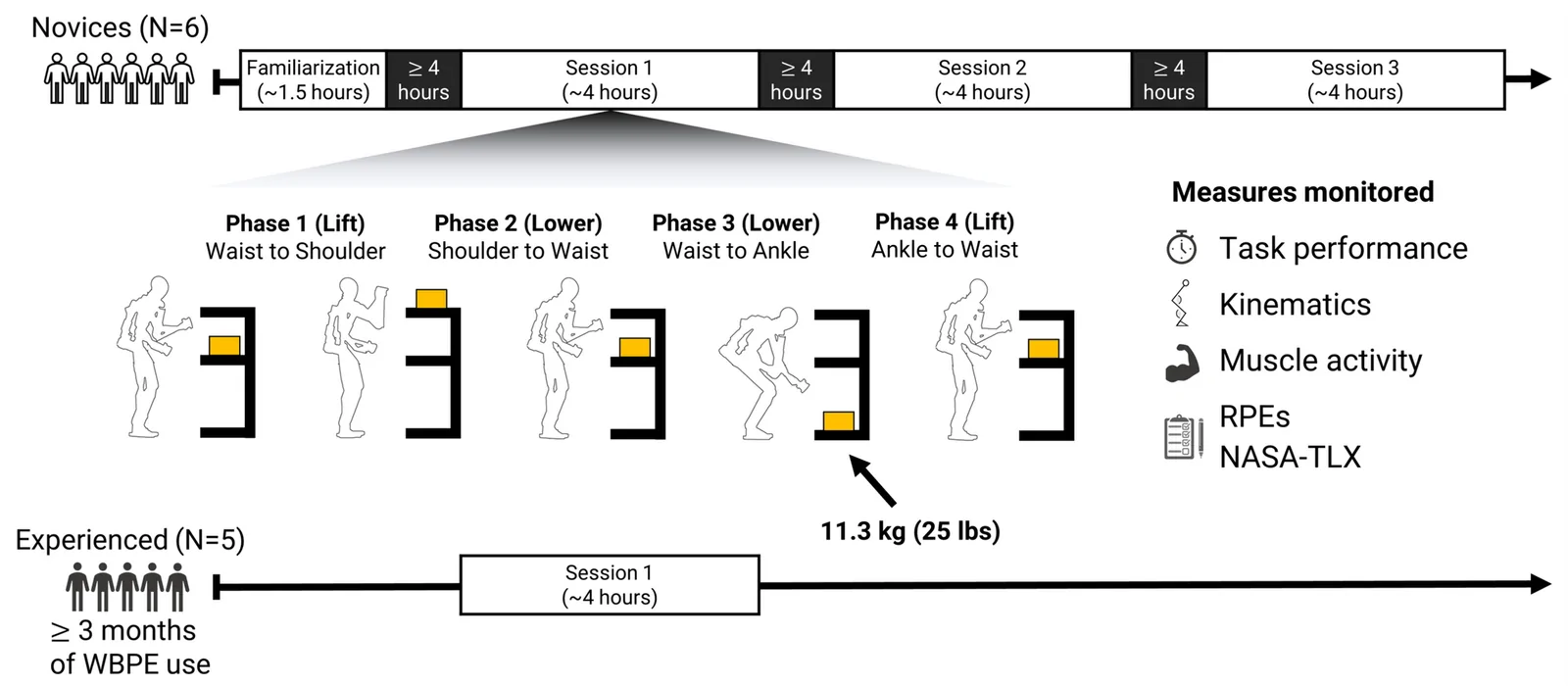
Overview of the experimental protocol. Novices completed one familiarization session with the whole-body powered exoskeleton (WBPE) followed by three load-handling sessions, while experienced users completed one load-handling session (the extended arrow for experienced users is shown only to align with the novice timeline and does not indicate additional sessions)

Novices completed four sessions with the exoskeleton and one control session without it (Fig. 2). The first session (∼1.5 h) provided familiarization and exoskeleton parameter tuning. Participants were trained in basic operations (donning/doffing, safety protocols, fitting). For fitting, participants first stepped into the footplates and secured an adjustable harness (shoulder/chest straps, hip and waist belts). They then completed a graded training protocol in which they initially operated only the upper-arm module to perform lifts, walked a 10-m gait track with and without loads, and practiced transferring loads from ankle to shoulder to experience a full joint range of motion. Tunable control parameters of the exoskeleton (e.g., actuation gains, payload, and gravity compensation) were adjusted throughout these exercises, with assistance from manufacturer training personnel.

The three subsequent load-handling sessions (S1–S3, ~4 h each) involved simulated occupational tasks (e.g., walking, load carriage, cart pushing, load handling). Only load-handling results are reported here (results from the walking task were reported in our previously noted publication). In each session, participants transferred an 11.3 kg (sandbag with handles) between three rack levels (11, 103, 168 cm), approximating foot, elbow, and head heights of an average U.S. adult [30], using their dominant arm. Participants stood roughly one arm’s length from the rack and were instructed to keep their foot positions as fixed as possible to simulate stationary work. Minor adjustments were permitted to reach the bottom shelf. Each trial began with the bag on the middle shelf and comprised four sequential lift/lower phases (Fig. 2). Participants completed two trials per session, which were self-paced and with short rest periods provided between trials if needed to minimize fatigue. The 11.3 kg load was chosen since our previous work [23] indicated that loads exceeding approximately 10 kg yielded beneficial effects of the whole-body powered exoskeleton (i.e., reduced activity of the back and leg muscles). Sessions were spaced ≥ 4 hours apart, all within four days to minimize fatigue and loss of adaptation. Novices completed one control condition (no exoskeleton) during either Session 1 or Session 3. Experienced users completed the control condition and one load-handling session with the exoskeleton on the same day.

### Data Collection

Whole-body kinematics were sampled at 60 Hz using a 17-sensor inertial measurement unit (IMU) system (MTW Awinda, Xsens Technologies B.V., Enschede, The Netherlands). Joint kinematics and 3D coordinates of anatomical landmarks were derived in MVN Animate using the ISB-recommended Euler sequence [31].

Muscle activity in the trunk, legs, and the dominant arm was monitored at 1.5 kHz using a telemetered surface electromyographic (EMG) system (Ultium, Noraxon, AZ, USA). For novices, muscle activity was only measured during Session 1 and Session 3, mainly because participants had limited availability for data collection. Initial skin preparation included shaving and cleaning with alcohol. Pairs of pre-gelled, bipolar, Ag/AgCl electrodes with a 2.5 cm inter-electrode spacing were placed bilaterally over the lumbar erector spinae (LES), and unilaterally (dominant side) over the anterior deltoid (AD), vastus lateralis oblique (VL), and biceps femoris (BF), based on procedures described previously [32]. For EMG normalization, participants performed maximum voluntary isometric contractions (MVICs) three times for each muscle group. LES contractions were obtained as participants stood with their trunk flexed to ∼20°, with their feet slightly separated, and they were asked to perform maximal trunk extension while their pelvis and legs were secured to a rigid fixture. For the AD, participants were asked to raise their dominant arm as hard as possible, while the arm was held at approximately 60°, 90°, and 120° shoulder flexion, with resistance provided by an investigator [33]. MVICs for the leg were performed as participants sat on a chair with their knee joint angle maintained at ∼90°, and they attempted knee and ankle flexion and extension against manual resistance [34]. A minimum of 30-second rest was given between MVICs.

Subjective responses were obtained regarding perceived demands. To assess physical demands, participants provided ratings of perceived exertion (RPEs) using the Borg CR–10 scale [35], with unilateral ratings for the shoulder and bilateral ratings for back and legs. To assess cognitive workload, unweighted NASA-TLX scores were recorded across six workload dimensions [36]: mental, physical, temporal (time) demands, performance, effort, and frustration. All subjective responses were obtained at the end of each session.

### Data Processing and Outcome Measures

All objective measures were computed separately for the four task phases: task completion time, movement smoothness, sagittal-plane joint kinematics, and muscle activity. For each phase, task completion time was the elapsed time from the instant the load was lifted off the initial rack level to the instant it was placed on the target rack level. Movement phases were segmented based on task events identified from the dominant hand vertical velocity profiles to identify lift onset, transitions between rack levels, and load placement, with precise phase boundaries visually confirmed using MVN Animate (Xsens Technologies). Movement smoothness was quantified using a jerk index (JI), which is a common metric for quantifying the movement smoothness of the 3D hand trajectory (e.g., [37–39]). JI was used as a primary indicator of coordination, because smoother hand trajectories reflect more efficient and stable human–exoskeleton interaction, with higher JI associated with increased corrective actions. Note that coordination was interpreted more broadly using JI in combination with task performance, joint kinematics, and muscle activity measures. A normalized JI was obtained, which supports comparisons of motions across varying time scales due to its scale-independent nature [40], and was calculated by taking the natural logarithm of the dimensionless integrated squared jerk:

1 $${\mathrm{JI}}\; = \;\ln \left( {\frac{{\left( {t_{2} - t_{1} } \right)^{5} }}{{D^{2} }}\mathop \smallint \limits_{{t_{1} }}^{{t_{2} }} \left( {\dddot x\left( t \right)^{2} + \dddot y\left( t \right)^{2} + \dddot z\left( t \right)^{2} } \right)dt} \right),$$

where $\dddot{x}(t)$, $\dddot{y}(t)$, and $\dddot{z}(t)$ are jerk components of the dominant hand in the lateral (x), anterior–posterior (y), and vertical (z) directions, respectively; these were obtained as the third derivatives of the position signals x(t), y(t), and z(t); *D* is the 3-D path length of the dominant hand; and ${t}_{1}$ and ${t}_{2}$ are the movement start and end times. Since the exoskeleton end-effector was not instrumented with an IMU, hand movement of the exoskeleton was estimated using the dominant hand IMU, which moved synchronously with the end-effector (Fig. 1).

Velocity, acceleration, and jerk time series were obtained by successive numerical differentiation of the position signals using a five-point central difference method [41]. The 3D path length *D* was computed as the integral of tangential velocity $V\left(t\right)$ over the movement duration:

2 $$D={\int }_{{t}_{1}}^{{t}_{2}}V\left(t\right) dt ,$$

where the tangential velocity $V\left(t\right)$ was defined as:

3 $$V\left(t\right)=\sqrt{{\left(\frac{dx}{dt}\right)}^{2}+{\left(\frac{dy}{dt}\right)}^{2}+{\left(\frac{dz}{dt}\right)}^{2}} ,$$

Joint angles and angular velocities were computed for the dominant shoulder, trunk, and dominant hip in the sagittal plane. Segment orientations of the upper arm, trunk, pelvis, and upper leg were obtained from their respective IMUs. Shoulder joint angle was calculated as the angle between the upper arm and trunk; trunk flexion as the angle of the trunk segment relative to the vertical axis; and hip joint angle as the included angle between the pelvis and upper leg. These measures were chosen to align with prior exoskeleton studies, which commonly analyzed trunk flexion and shoulder elevation for their relevance to physical demands and exoskeleton support [42, 43].

Raw EMG signals were band-pass filtered (20–450 Hz, 4th-order Butterworth, bidirectional), rectified, and low-pass filtered (3 Hz cut-off, 4th-order Butterworth, bidirectional) to create linear envelopes. Linear envelopes for each of the four muscle groups were normalized to corresponding peak values obtained during MVICs to obtain normalized EMG (NEMG) envelopes. Then, 5th, 50th, and 95th percentile values were computed using the empirical cumulative distribution function (ECDF), representing low-level, median, and near-peak activation levels, respectively. These metrics were computed specifically for the dominant side LES (LES_DOM_), VL (VL_DOM_), BF (BF_DOM_), AD (AD_DOM_), non-dominant side LES (LES_NDOM_), VL (VL_NDOM_), and BF (BF_NDOM_). ECDF-based percentile metrics were selected because they are commonly used to characterize low, typical, and near-peak muscle demands during occupational tasks, enabling comparisons across the entire lifting and lowering phases [44, 45].

### Statistical Analyses

Linear, mixed-effects models (LMMs) were fitted for each outcome measure to compare each novice exoskeleton session with the single experienced exoskeleton session. For task completion time, movement smoothness, muscle activity, and joint kinematics, *Session* and *Phase* were included as fixed effects, and participants were modeled as random effects. *Session* was treated as a four-level categorical factor, representing the three novice sessions and the experienced user session. *Phase* was treated as a four-level within-subject categorical factor (four task phases). Model comparisons employed likelihood ratio tests, contrasting a full model with *Session* × *Phase* interaction term against a reduced model with only main effects of *Session* and *Phase*. If the interaction term was significant, the full model was retained for subsequent *post hoc* analyses; otherwise, the reduced model was used. For subjective measures (RPEs and NASA-TLX), separate LMMs were fitted without including *Phase*. No substantial violations of parametric model assumptions were evident. All analyses were conducted using JMP Pro 18.0 (SAS Institute Inc., Cary, NC), with statistical significance set at *p* < 0.05. Full model parameters, test statistics, and *p*-values for all outcome measures are reported in the Supplementary Information (Tables S1–S4). Given the small sample size and exploratory scope, *post hoc* paired comparisons were done using *t-*tests without multiple-comparison adjustments.

## Results

### Task Performance and Movement Smoothness

**Fig. 3:**
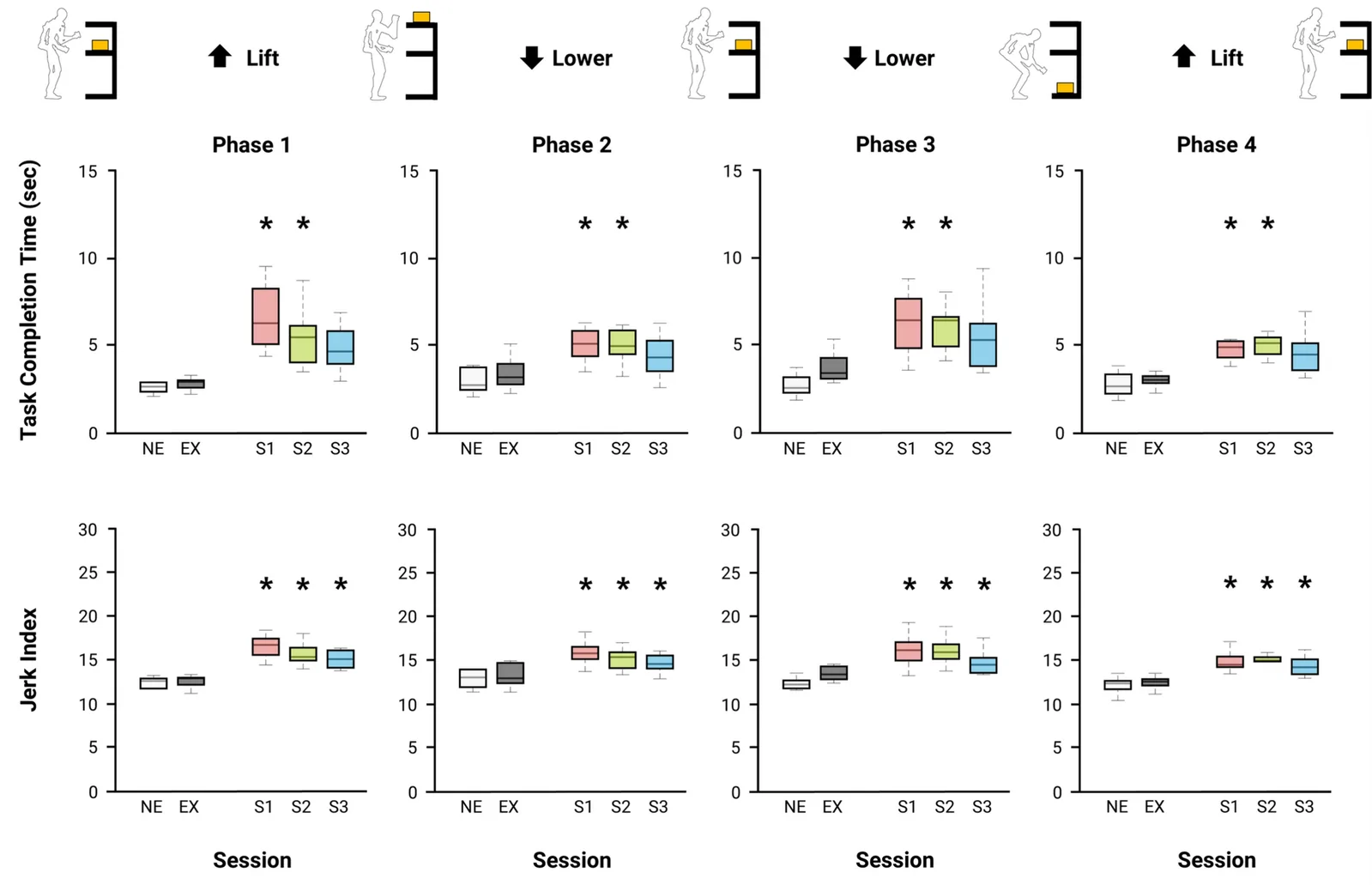
Mean task completion time (first row) and jerk index (JI; second row). Box plots summarize the results when using the exoskeleton for novice Session 1 (S1; red), Session 2 (S2; green), and Session 3 (S3; blue), and the experienced users (EX; black). For context, the white plot summarizes the results from the no-exoskeleton session (NE) across participants in both groups. The symbol * indicates a significant *Session* effect between the corresponding novice session and the experienced user session

There was a significant main effect of *Session* on both task completion time and JI, but the *Session* × *Phase* interaction was not significant for either variable (Table S1 in the Supplementary Information). Novices differed most from experienced users in Session 1, with differences decreasing across subsequent sessions (Fig. 3). Specifically, completion time was significantly longer for novices in Session 1 and Session 2 (up to ~117% longer in Session 1 compared to the experienced group), reducing to about 59% longer than the experienced group in Session 3. Similarly, JI was significantly larger for novices than experienced users across all three sessions, with the largest difference in Session 1 (~27% difference with the experienced group) and smallest difference in Session 3 (~19%). Differences in JI were more pronounced during the lifting phases (1 and 4) compared to the lowering phases (2 and 3), with significant differences persisting through Session 3.

### Joint Kinematics

**Fig. 4:**
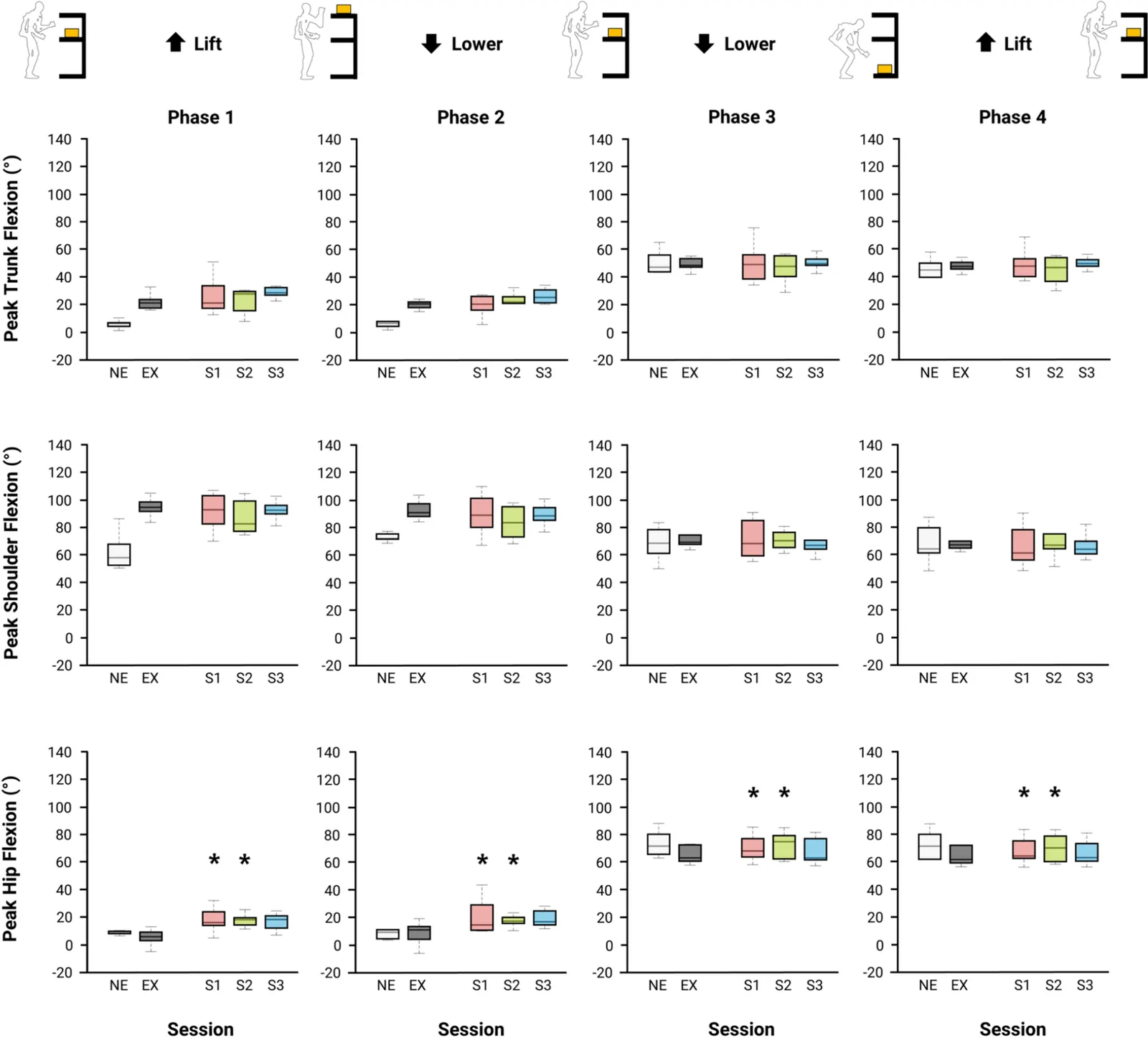
Mean peak trunk flexion (first row), peak shoulder flexion (second row), and peak hip flexion (third row) across *Session* and *Phase*. Box plots summarize the results when using the exoskeleton for novice Session 1 (S1; red), Session 2 (S2; green), and Session 3 (S3; blue), and the experienced users (EX; black). For context, the white plot summarizes the results from the no-exoskeleton session (NE) across participants in both groups. The symbol * indicates a significant *Session* effect between the corresponding novice session and the experienced user session

Only peak hip flexion showed a significant main effect of *Session*, with no significant *Session* × *Phase* interactions for any joint angle measures (Fig. 4 and Table S2 in the Supplementary Information). Novices used significantly greater hip flexion than experienced users during Session 1 and Session 2, with differences up to ~13° (235% increase compared to the experienced group). In contrast, peak trunk and shoulder flexion showed no significant main effects of *Session*, although both measures varied substantially across lifting phases. Novices used approximately 7° (23%) greater trunk flexion when transferring the load between waist and shoulder heights (phases 1 and 2) and ~8° (9%) smaller peak shoulder flexion compared to the experienced group, but these differences were not statistically significant.

**Fig. 5:**
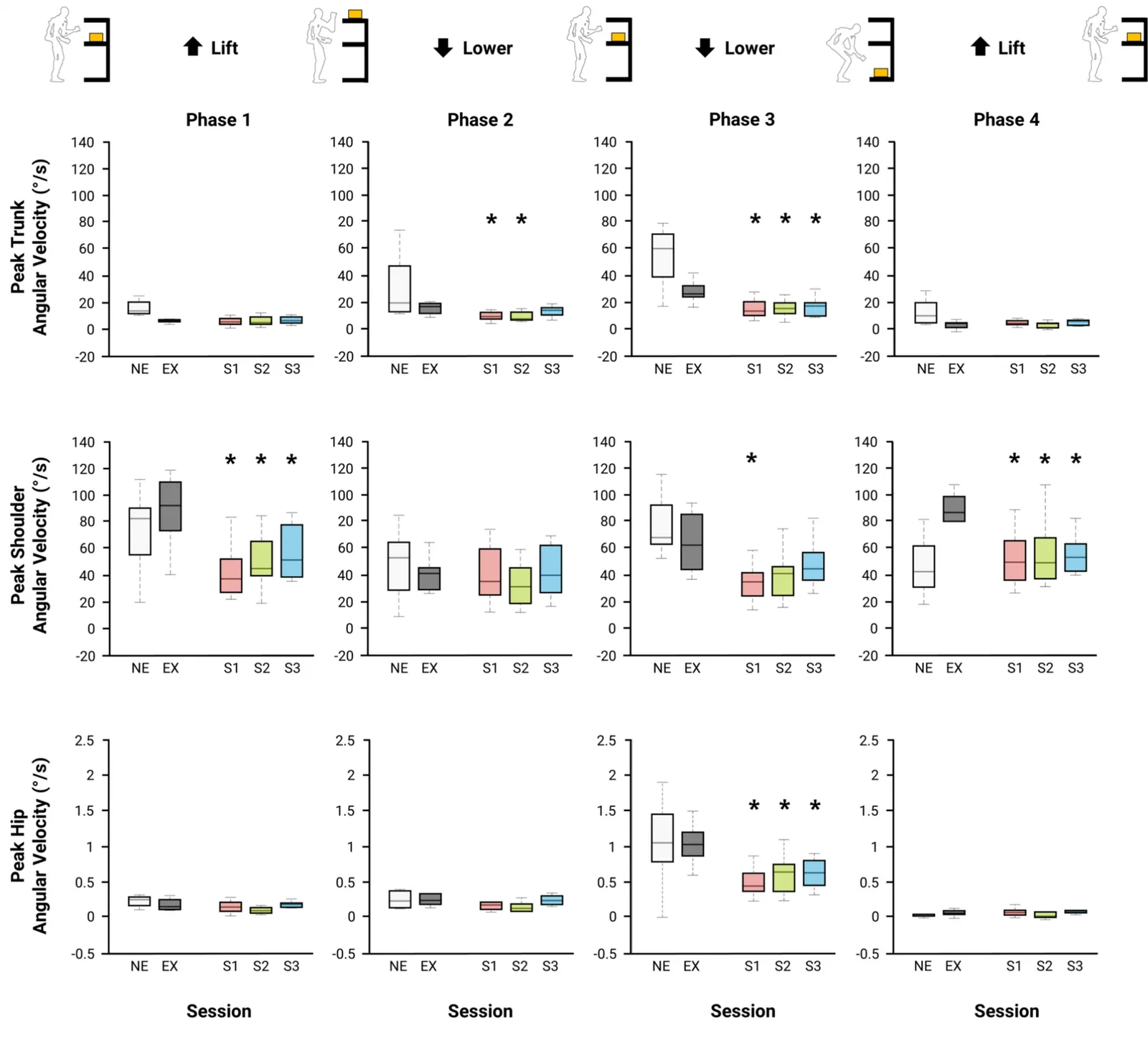
Mean peak angular velocity of trunk flexion (first row), shoulder flexion (second row), and hip flexion (third row). Box plots summarize the results when using the exoskeleton for novice Session 1 (S1; red), Session 2 (S2; green), and Session 3 (S3; blue), and the experienced users (EX; black). For context, the white plot summarizes the results from the no-exoskeleton session (NE) across all participants in both groups. The symbol * indicates a significant paired difference between the corresponding novice session and the experienced user session within the given phase

There were more substantial and consistent differences in angular velocities between novices and experienced users (Fig. 5 and Table S2 in the Supplementary Information), with significant *Session* × *Phase* interaction effects for peak trunk, shoulder, and hip angular velocities. Novices flexed their trunks and hips significantly slower when lowering the load from waist to ankle level (phase 3), with respective differences of up to ~14°/s (48%) and 32°/s (52%). These slower movements remained through Session 3. During lifting (phases 1 and 4), novices also elevated their arms significantly slower, by up to ~47°/s (53%). Although the difference in shoulder angular velocities between novices and experienced users decreased over sessions, novices remained slower at Session 3.

### Muscle Activity

**Fig. 6:**
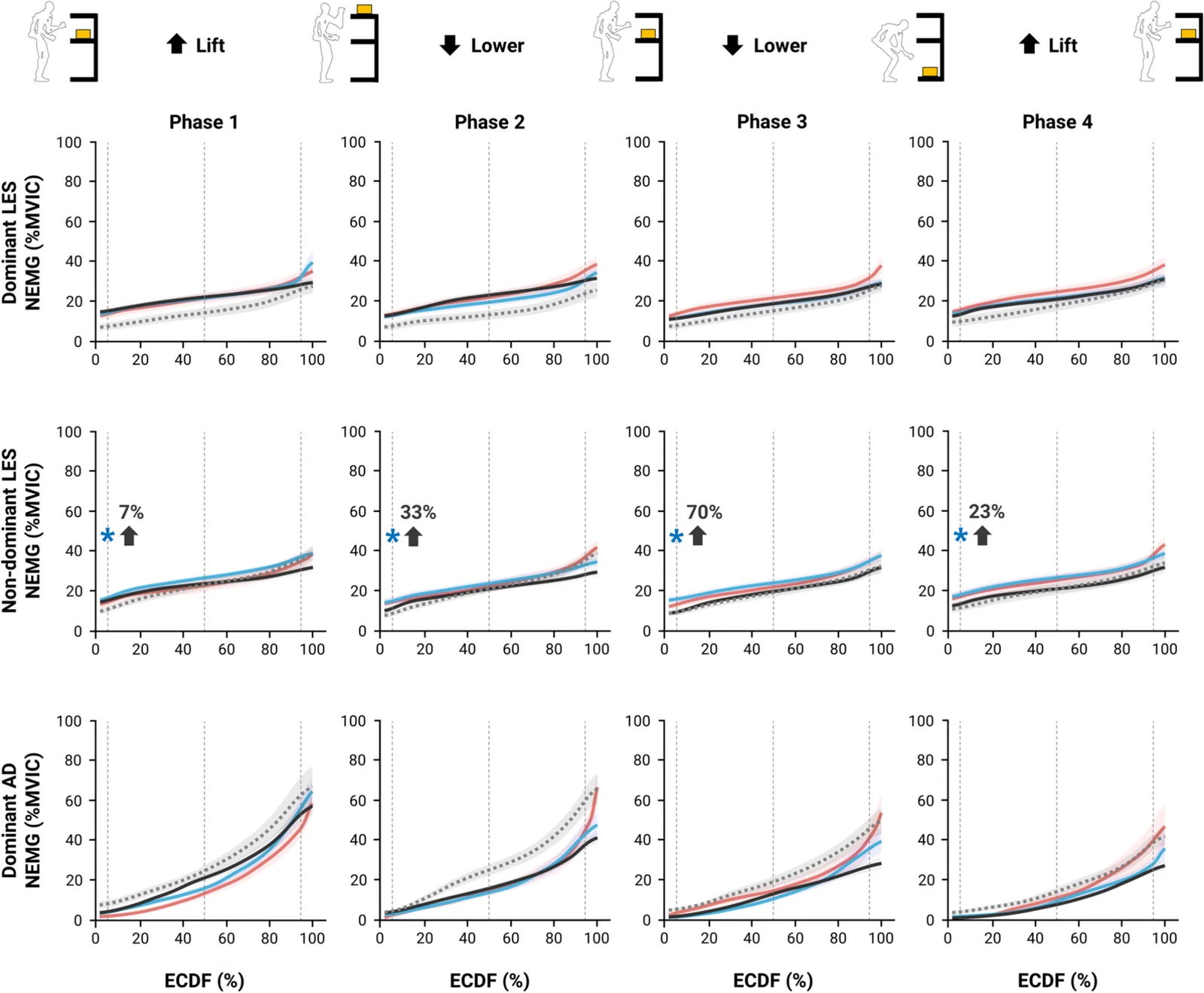
Activity levels in the dominant lumbar erector spinae (LES; first row), non-dominant lumbar erector spinae (second row), and dominant anterior deltoid muscle (AD; third row). Each plot represents the ECDF of muscle activity. Solid lines indicate mean muscle activity in novice Session 1 (red) and Session 3 (blue), and among the experienced users (black). The dashed black line represents muscle activity of the no-exoskeleton session (NE) across all participants. Colored regions indicate ± 2 standard errors for each corresponding phase of the load transfer tasks. Vertical dashed lines intercept the 5th, 50th, and 95th percentile values of each ECDF. An asterisk (*) indicates a significant *Session* effect between a given novice session and the experienced session at that percentile, and the color of the asterisk denotes the novice session in which the difference was observed. Arrows indicate the percentage difference between novice and experienced sessions

Differences in muscle activation patterns between novice and experienced users were generally modest (Fig. 6 and Table S3 in the Supplementary Information), especially for back and shoulder muscles. No significant *Session* × *Phase* interaction effects were found, and a significant main effect of *Session* was only found for the 5th percentile of LES_NDOM_ in Session 3. In the latter case, novices exerted 29% (3.8% MVIC) higher activity than experienced users. Interestingly, though not statistically significant, mixed results were found for the AD_DOM_: novices had lower 5th and 50th percentile levels of muscle activity (up to 14%) but higher 95th percentile values (up to 22%) compared to experienced users.

**Fig. 7:**
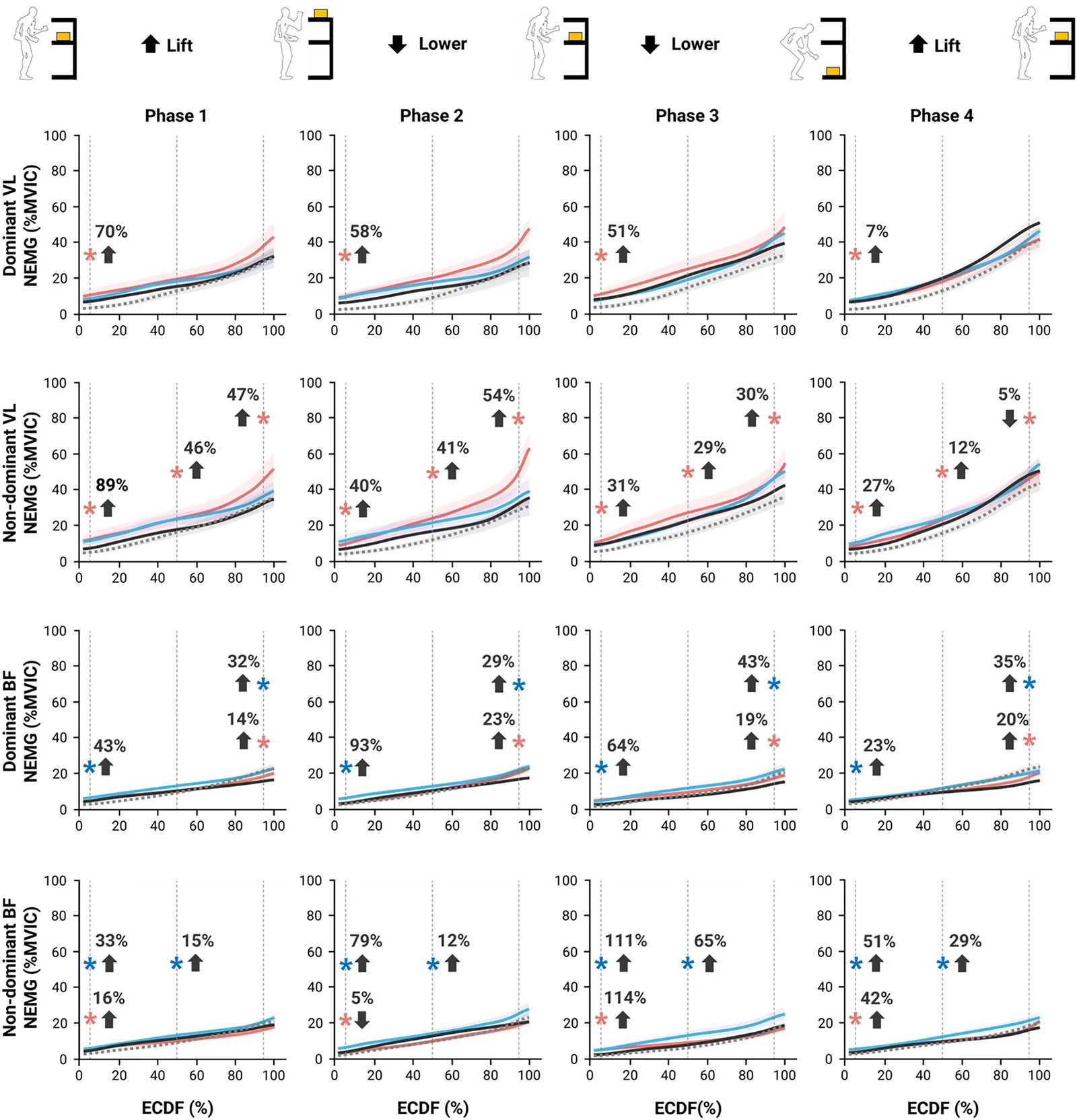
Activity levels in the dominant vastus lateralis (VL; first row), non-dominant vastus lateralis (second row), dominant biceps femoris (BF; third row), and non-dominant biceps femoris muscle activity (fourth row). Each plot represents the ECDF of muscle activity. Solid lines indicate mean muscle activity in novice Session 1 (red) and Session 3 (blue), and among the experienced users (black). The dashed black line represents muscle activity of the no-exoskeleton session (NE) across all participants. Colored regions indicate ± 2 standard errors for each corresponding phase of the load transfer tasks. Vertical dashed lines intercept the 5th, 50th, and 95th percentile values of each ECDF. An asterisk (*) indicates a significant *Session* effect between a given novice session and the experienced session at that percentile, and the color of the asterisk denotes the novice session in which the difference was observed. Arrows indicate the percentage difference between novice and experienced sessions

Activation patterns in the leg muscles showed more distinct differences between novice and experienced users (Fig. 7 and Table S3 in the Supplementary Information). Significant main effects of *Session* were observed for several outcomes, particularly during Session 1, during which novices exhibited higher muscle activation across multiple measures. Specifically, for the VL, novices had significantly larger 5th percentile activity on both the dominant and non-dominant sides during Session 1, by up to ~46% when compared to the experienced group (i.e., 3.2% MVIC). Similarly, significant main effects were found for the 5th, 50th, and 95th percentiles of VL_NDOM_ in Session 1, by up to ~28% (11% MVIC). For the BF, there were significant *Session* effects on the 5th percentile (dominant side in Session 3; non-dominant side in Session 1 and Session 3) and the 50th percentile (non-dominant in Session 3). At the 95th percentile, dominant side activation was significantly higher among novices in both Session 1 and Session 3. These findings indicate that novices tended to sustain higher levels of muscle activation throughout the tasks, particularly for the BF, with differences of up to ~34% (5.6% MVIC) on the BF_DOM_ and ~27% (2.9% MVIC) on the BF_NDOM_ at certain percentiles.

### Subjective Ratings

**Fig. 8:**
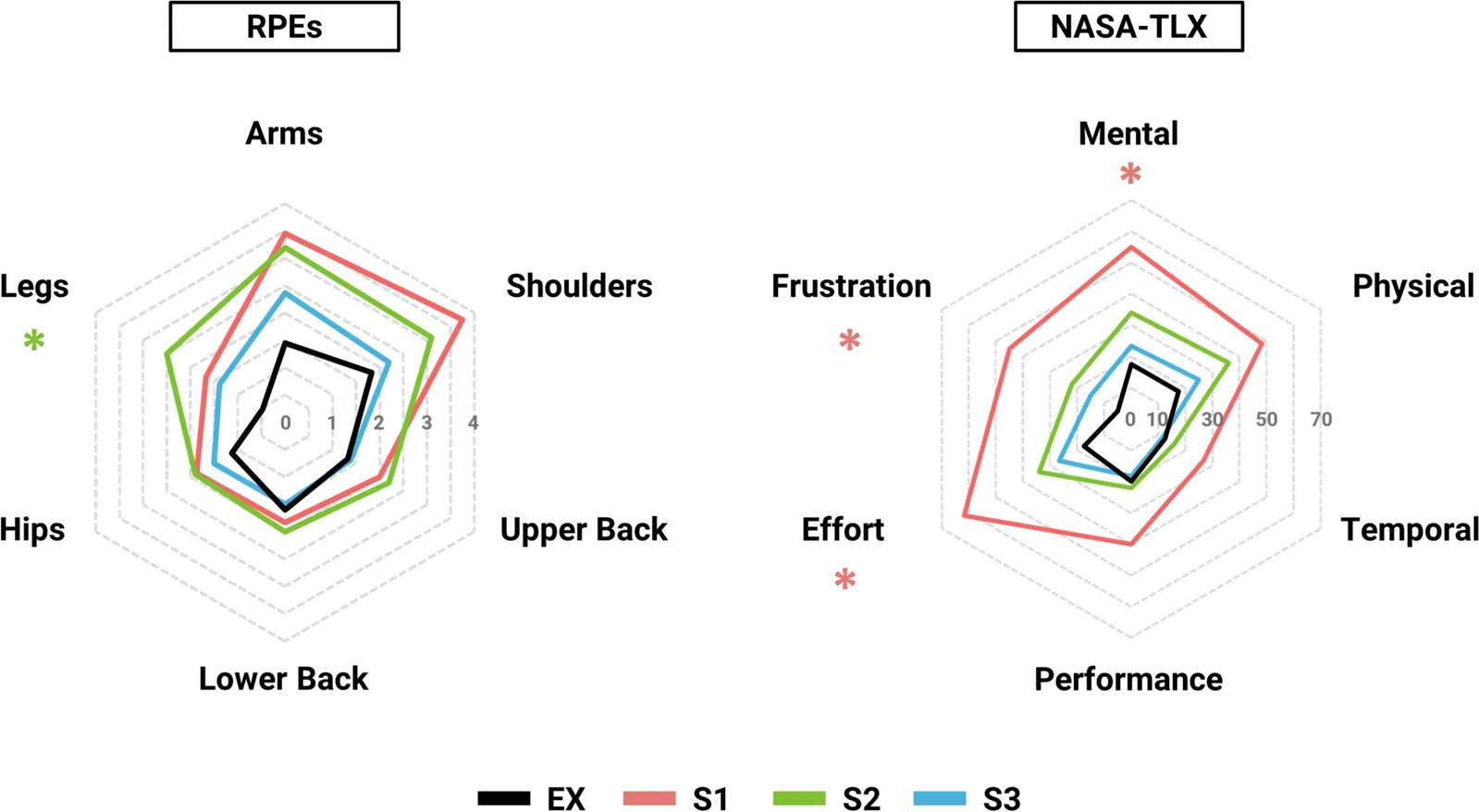
Mean RPEs (left) and NASA-TLX scores (right). Each radar chart represents mean values from the novice Session 1 (red), Session 2 (green), and Session 3 (blue), and the experienced users (black). The symbol * indicates a significant paired difference between the corresponding novice session and the experienced user session, with the symbol color indicating a specific experimental session

Novices generally reported greater perceived exertion and cognitive workload compared to experienced users during the initial sessions (Fig. 8 and Table S4 in the Supplementary Information). However, there were no statistically significant effects of *Session* for RPEs at the arm, shoulder, upper back, or lower back. Only RPE at the legs was significantly higher among novices during Session 2 compared to experienced users, but this difference was not sustained in Session 3. Novices reported significantly greater mental demand, effort, and frustration in Session 1 compared to experienced users. In particular, mental demand scores for novices were over twice as high as those for experienced users in Session 1 (~214% greater), decreasing to ~33% by Session 3. Frustration ratings were also significantly elevated in Session 1. These subjective workload differences typically decreased over subsequent sessions, resulting in no significant differences by Session 3. No significant differences were observed for physical demand, temporal demand, or performance ratings across sessions.

## Discussion

Our initial hypotheses were partially supported in that novices showed substantial improvements in task completion time and perceived workload, reaching levels comparable to experienced users by Session 3. In contrast, their hand trajectories remained less smooth than those of experienced users, confirming our expectation that movement smoothness would require extended training to achieve proficiency. We discuss these outcomes in more detail subsequently.

### Task Completion Time and Movement Smoothness

There were pronounced initial differences between novices and experienced users in task completion time and movement smoothness (JI; Fig. 3). Novices were approximately 117% slower and 27% less smooth in their hand movements than experienced users in Session 1, although they did improve over time (ending at 59% slower and 19% less smooth by Session 3). The absence of *Session* × *Phase* interactions for either of these measures suggests that adaptation appears to reflect a global change in strategy rather than adjustments specific to different lifting phases. Nevertheless, novices still exhibited performance differences compared to experienced users after three training sessions, which suggests that they had not yet formed the internal models needed to proficiently operate the whole-body powered exoskeleton [26, 46]. This outcome aligns with our earlier gait study using the same exoskeleton, in which novices continued walking with shorter steps and slower speed after three training sessions [25]. Though a direct comparison is not feasible due to differences in study design (between- vs. within-subject testing), comparable decreases in task completion time were also reported for a simpler powered exoskeleton during various functional tasks (e.g., load carrying or stair climbing), relative to performing the tasks without an exoskeleton [12].

That novices here had significantly higher JI than experienced users implies that they had not yet reached an efficient, minimum-jerk movement pattern. Since JI is sensitive to abrupt corrections and motor noise, the persisting gap between novices and experienced users is likely due to the additional cognitive load of coordinating the complex whole-body powered exoskeleton inertia along with sensorimotor uncertainty at the human–exoskeleton interface. As Wilkenfeld et al. [47] noted, it is also likely that novices were engaging in *sensemaking* strategies, such as adapting their approach or reframing the exoskeleton as a tool, to better align their actions with the device. The elevated JI we observed among novices is consistent with this interpretation, indicating the abrupt corrections and exploratory adjustments required while establishing coordination with the whole-body powered exoskeleton. Evidence from previous motor adaptation studies further supports this interpretation within established motor learning frameworks. Novice climbers, for instance, had the highest JI on their first attempt at a new route, which then dropped sharply as technique stabilized [48], and similar early spikes in jerk and variability have been reported in wrist cursor training [49] and roller-ball learning [50]. Although these studies did not involve exoskeletons, they demonstrate that elevated jerk is a consistent hallmark of early feedback-dominated control, suggesting ongoing exploration and error correction in unfamiliar dynamic environments. As predictive internal models are formed, movements become smoother and more feed-forward in nature, leading to reductions in JI. Interpreted in this context, the persistently higher JI observed among novices suggests incomplete internal model formation for the coupled human–exoskeleton system after three sessions. Future work should include within-subject exoskeleton vs. no-exoskeleton comparisons to more fully capture the adaptation trajectory of novice users.

### Joint Kinematics and Muscle Activity

Although novices showed some progression over sessions, indicating they were adapting to perform more like the experienced users, they consistently leaned forward more than experienced users. Such a forward-flexed posture parallels our earlier findings during gait [25], in which novices reported self-selecting this strategy to lower the whole-body center-of-mass (COM) and feel more stable while adapting to balance the whole-body powered exoskeleton [47]. From the perspective of the body as an inverted pendulum [51], elevating the COM increases postural sway and the challenge of maintaining balance. Park et al. [52] noted that using a passive back-support exoskeleton shifted the COM superiorly, but the context differs from the present whole-body powered exoskeleton. In our case, control of balance was primarily governed by the user’s interaction with the actuated structure, and the system’s bulk may have contributed to shifts in COM. However, because we did not directly measure COM, it is unclear whether the COM was elevated. Regardless, vertical shifts in COM would have occurred during load transfers across different height levels, and novices may have compensated by using greater hip flexion to enhance perceived stability, while also being limited in ankle range of motion due to the flat metal footplate [25].

Novices used greater activation of the bilateral VL and BF, whereas back and shoulder activations differed little from experienced users. We suspect that the former resulted from novices stiffening their lower limbs to reduce postural sway. Such compensation improves balance control when sensory prediction is uncertain, though passive muscle stiffness alone may not account for sway control [53, 54]. In contrast, muscle activity of the back and shoulder did not differ substantially between novices and experienced users, despite novices rotating their trunk and upper arm more slowly. Prior work suggests that the velocity–activation relationship is task-dependent [55, 56], and may be further complicated by whole-body movements with the added mass of the exoskeleton. Our results therefore indicate that novices produced similar muscle activity despite moving more slowly, suggesting a possible strategic trade-off. Experienced users achieved faster and smoother movements (lower JI), reflecting greater coordination and control. By comparison, novices may have compensated for limited control by moving more slowly, minimizing muscle effort but sacrificing performance. It appears that three training sessions were not sufficient for novices to achieve the skilled coordination and efficiency demonstrated by experienced users.

### Perceived Exertion and Cognitive Workload

Unlike several objective measures that continued to differ between novices and experienced users across sessions, subjective ratings converged more quickly. Novices initially reported increased perceived exertion and cognitive workload when using the whole-body powered exoskeleton, whereas experienced users reported low ratings in both domains. Across body regions, RPEs did not differ significantly between novices and experienced users except for a transient increase in the legs in Session 2. By Session 3, both RPE and NASA-TLX scores among novices had declined to levels comparable to those of experienced users, indicating substantial adaptation. This temporal pattern supports earlier reports of initial physical and cognitive challenges when first using a whole-body powered exoskeleton [47]. Similarly, Bequette et al. [57] found that short-term use of powered lower-limb exoskeletons led to increased NASA-TLX ratings for mental, physical, effort, and frustration domains, emphasizing the cognitive-emotional demands associated with powered exoskeleton use. Findings from these previous studies, which identified initially elevated cognitive workload when using powered exoskeletons, suggest that perceived workload during early exoskeleton use may be influenced predominantly by cognitive and affective factors, rather than muscular exertion alone. Accordingly, structured familiarization protocols that enhance user confidence and clarify device response characteristics may be important for minimizing initial cognitive effort and supporting efficient adaptation in human–exoskeleton interaction.

## Limitations and Future Work

Some limitations of the current study should be noted. First, the sample size was relatively small and included only male participants. Recruitment of participants with relatively consistent anthropometric measures was necessary so that experimental participants could fit within the exoskeleton prototype. To generalize our findings, broader samples should be considered in the future, with different age groups, sexes, and anthropometric characteristics. Second, although we identified experienced users based on consistent criteria (i.e., more than 3 months of exposure) and subjective opinions, it remains an open research question regarding how to define whole-body powered exoskeleton expertise more precisely and objectively. Third, motor adaptation was evaluated over only three brief sessions. Thus, novices may not have reached their optimal performance within the assessment period. Finally, the prototype whole-body powered exoskeleton we used was still in the developmental phase.

Several directions for future research follow from these limitations. Longer-term training and retention testing are needed to characterize the full time course of adaptation, including the transition from feedback-dominated exploration to more stable feed-forward control. Future work should also examine how structured familiarization protocols influence adaptation outcomes. For example, a recent study implemented a multi-week familiarization protocol grounded in motor learning principles for an adjustable passive load-bearing exoskeleton during loaded walking [58]. Notably, familiarization did not uniformly reduce metabolic cost or muscle activation, highlighting that “longer exposure” alone may be insufficient and that individual responses can vary. Extending such structured familiarization approaches to whole-body powered exoskeletons could help identify which elements of familiarization (e.g., task selection, progression, feedback) most strongly affect the emergence of feed-forward control.

At the same time, future studies should more explicitly incorporate user-centered perspectives to guide both testing and design iteration. For instance, prior work has described the development and preliminary performance assessment of a modular full-body exoskeleton using a user-centered design approach that integrated end-user needs, biomechanical characterization, and usability evaluation [59]. Similar frameworks could be applied to whole-body powered exoskeleton prototypes to better align task demands, interface design, and control behavior with user capabilities, potentially reducing sensorimotor uncertainty at the human–exoskeleton interface.

Finally, results from multiple studies suggest that adaptation to exoskeletons is highly individualized (e.g., [60, 61]). Accordingly, future work should evaluate predictors of adaptation rate and performance (e.g., physical capacity, prior experience, movement strategy, comfort), with the goal of developing personalized familiarization approaches that match training structure and device settings with individual user characteristics.

## Conclusion

Across three sessions, novices underwent substantial, though apparently incomplete, motor adaptation when using a pre-alpha whole-body powered exoskeleton for stationary load handling. Although the tasks we simulated did not require maintaining dynamic postural stability (as in gait or locomotor tasks), novices were still unable to achieve motor coordination comparable to experienced users, particularly in task performance aspects. Differences in joint kinematics and muscle activity between novices and experienced users diminished more rapidly than the persistent gaps in task completion time and JI. However, novices continued to exhibit slower trunk, shoulder, and hip angular velocities. Consistent with our earlier findings during gait tasks, novices generally employed a more trunk flexed posture, potentially to maintain their COM in a more inferior position and enhance overall balance. While preliminary, these findings contribute to a better understanding of the motor control and adaptation strategies exhibited by users of whole-body powered exoskeletons, guide future design developments, and formulate training guidelines for the practical implementation of this technology.

## Supplementary Information

Below is the link to the electronic supplementary material.Supplementary file1 (DOCX 65 kb)

## Data Availability

The datasets are not publicly available because some contain identifiable information but are available from the corresponding author on reasonable request. Requests to access the datasets should be directed to sriniv5@clemson.edu.
