# Supplementary material for: Adaptation to a Whole-Body Powered Exoskeleton: Human–Exoskeleton Coordination During Load-Handling Tasks
Source: Ann Biomed Eng. 2026 Mar 10;54(9):3109–23. doi: 10.1007/s10439-026-04025-9 (PMC13522028; doi:10.1007/s10439-026-04025-9)
Supplement: Supplementary file 1 [file 10439_2026_4025_MOESM1_ESM.docx]

**Supplementary Information**

Table S1. Linear mixed-effects model (LMM) parameters for main effects of *Session* and *Phase* on task completion time and JI in stationary load handling tasks, analyzed separately by dependent measures*.* All models included the *Phase* × *Session* interaction term; however, likelihood ratio tests (LRT) indicated that these interactions were not significant for task completion time or JI; therefore, interpretations focused on the main effects. Statistically significant *p*-values are indicated in bold.

| **Dependent**  **Measures** | **Fixed Effect** | **Parameter**  **Estimate** | **Std.**  **Error** | ***t* value** | ***p* value** |
| --- | --- | --- | --- | --- | --- |
| *Task Completion*  *Time (sec)* | Intercept | 3.44 | 0.56 | 6.19 | **<0.001** |
|  | Session 1 | 2.83 | 0.73 | 3.87 | **0.002** |
|  | Session 2 | 2.08 | 0.75 | 2.77 | **0.014** |
|  | Session 3 | 1.40 | 0.75 | 1.86 | 0.083 |
|  | Phase 2 | -0.32 | 0.22 | -1.45 | 0.149 |
|  | Phase 3 | 0.40 | 0.22 | 1.78 | 0.077 |
|  | Phase 4 | -0.63 | 0.22 | -2.84 | **0.005** |
| *Jerk Index* | Intercept | 13.24 | 0.42 | 31.18 | **<0.001** |
|  | Session 1 | 2.94 | 0.55 | 5.33 | **<0.001** |
|  | Session 2 | 2.48 | 0.57 | 4.37 | **<0.001** |
|  | Session 3 | 1.61 | 0.57 | 2.85 | **0.013** |
|  | Phase 2 | -0.21 | 0.19 | -1.09 | 0.280 |
|  | Phase 3 | 0.07 | 0.19 | 0.39 | 0.698 |
|  | Phase 4 | -0.79 | 0.19 | -4.14 | **<0.001** |

Table S2. Linear mixed-effects model (LMM) parameters for main effects of *Session* and *Phase* on joint kinematics in stationary load handling tasks, analyzed separately by dependent measures*.* All models included the *Phase* × *Session* interaction term; however, likelihood ratio tests (LRT) indicated that these interactions were not significant for joint kinematics; therefore, interpretations focused on the main effects. Statistically significant *p*-values are indicated in bold.

| **Dependent**  **Measures** | **Fixed Effect** | **Parameter**  **Estimate** | **Std. Error** | ***t* value** | ***p* value** |
| --- | --- | --- | --- | --- | --- |
| *Peak Trunk*  *Flexion* (°) | Intercept | 23.24 | 3.77 | 6.17 | **<0.001** |
|  | Session 1 | 2.79 | 5.05 | 0.55 | 0.590 |
|  | Session 2 | -1.10 | 5.19 | -0.21 | 0.834 |
|  | Session 3 | 2.09 | 5.19 | 0.40 | 0.692 |
|  | Phase 2 | -2.29 | 0.85 | -2.69 | **0.008** |
|  | Phase 3 | 24.48 | 0.85 | 28.73 | **<0.001** |
|  | Phase 4 | 23.65 | 0.85 | 27.75 | **<0.001** |
| *Peak Shoulder*  *Flexion* (°) | Intercept | 94.57 | 4.09 | 23.11 | **<0.001** |
|  | Session 1 | -2.69 | 5.44 | -0.50 | 0.629 |
|  | Session 2 | -3.55 | 5.61 | -0.63 | 0.537 |
|  | Session 3 | -3.33 | 5.61 | -0.59 | 0.562 |
|  | Phase 2 | -3.37 | 1.25 | -2.70 | **0.008** |
|  | Phase 3 | -20.75 | 1.25 | -16.66 | **<0.001** |
|  | Phase 4 | -24.42 | 1.25 | -19.60 | **<0.001** |
| *Peak Hip*  *Flexion* (°) | Intercept | 9.00 | 2.93 | 3.07 | **0.006** |
|  | Session 1 | 8.26 | 3.84 | 2.15 | **0.046** |
|  | Session 2 | 8.70 | 4.01 | 2.17 | **0.044** |
|  | Session 3 | 6.85 | 4.01 | 1.71 | 0.106 |
|  | Phase 2 | 1.50 | 1.21 | 1.23 | 0.219 |
|  | Phase 3 | 53.92 | 1.21 | 44.43 | **<0.001** |
|  | Phase 4 | 52.27 | 1.21 | 43.07 | **<0.001** |
| *Peak Trunk*  *Angular Velocity* (°/s) | Intercept | 7.10 | 1.66 | 4.28 | **<0.001** |
|  | Session 1 | -0.60 | 2.25 | -0.27 | 0.792 |
|  | Session 2 | -0.47 | 2.32 | -0.20 | 0.841 |
|  | Session 3 | 0.63 | 2.32 | 0.27 | 0.788 |
|  | Phase 2 | 8.67 | 1.76 | 4.92 | **<0.001** |
|  | Phase 3 | 21.55 | 1.76 | 12.22 | **<0.001** |
|  | Phase 4 | -3.00 | 1.76 | -1.70 | 0.091 |
|  | Session 1 × Phase 2 | -4.41 | 2.39 | -1.85 | 0.067 |
|  | Session 1 × Phase 3 | -13.18 | 2.39 | -5.52 | **<0.001** |
|  | Session 1 × Phase 4 | 2.10 | 2.39 | 0.88 | 0.380 |
|  | Session 2 × Phase 2 | -5.31 | 2.49 | -2.13 | **0.035** |
|  | Session 2 × Phase 3 | -12.34 | 2.49 | -4.95 | **<0.001** |
|  | Session 2 × Phase 4 | 0.53 | 2.49 | 0.21 | 0.833 |
|  | Session 3 × Phase 2 | -2.70 | 2.49 | -1.08 | 0.282 |
|  | Session 3 × Phase 3 | -11.99 | 2.49 | -4.81 | **<0.001** |
|  | Session 3 × Phase 4 | 1.21 | 2.49 | 0.48 | 0.629 |
| *Peak Shoulder*  *Angular Velocity* (°/s) | Intercept | 89.08 | 7.79 | 11.44 | **<0.001** |
|  | Session 1 | -47.10 | 10.54 | -4.47 | **<0.001** |
|  | Session 2 | -39.22 | 10.81 | -3.63 | **0.002** |
|  | Session 3 | -29.12 | 10.81 | -2.70 | **0.015** |
|  | Phase 2 | -47.29 | 5.30 | -8.92 | **<0.001** |
|  | Phase 3 | -24.83 | 5.30 | -4.69 | **<0.001** |
|  | Phase 4 | -4.87 | 5.30 | -0.92 | 0.360 |
|  | Session 1 × Phase 2 | 46.57 | 7.18 | 6.49 | **<0.001** |
|  | Session 1 × Phase 3 | 17.55 | 7.18 | 2.45 | **0.016** |
|  | Session 1 × Phase 4 | 16.07 | 7.18 | 2.24 | **0.027** |
|  | Session 2 × Phase 2 | 30.91 | 7.50 | 4.12 | **<0.001** |
|  | Session 2 × Phase 3 | 16.96 | 7.50 | 2.26 | **0.025** |
|  | Session 2 × Phase 4 | 9.45 | 7.50 | 1.26 | 0.209 |
|  | Session 3 × Phase 2 | 33.34 | 7.50 | 4.45 | **<0.001** |
|  | Session 3 × Phase 3 | 15.81 | 7.50 | 2.11 | **0.037** |
|  | Session 3 × Phase 4 | 3.72 | 7.50 | 0.50 | 0.621 |
| *Peak Hip*  *Angular Velocity* (°/s) | Intercept | 10.26 | 3.14 | 3.27 | **0.003** |
|  | Session 1 | -2.10 | 4.25 | -0.49 | 0.626 |
|  | Session 2 | -3.83 | 4.38 | -0.88 | 0.388 |
|  | Session 3 | 0.15 | 4.38 | 0.04 | 0.972 |
|  | Phase 2 | 7.40 | 3.37 | 2.20 | **0.030** |
|  | Phase 3 | 50.56 | 3.37 | 15.01 | **<0.001** |
|  | Phase 4 | -5.64 | 3.37 | -1.67 | 0.096 |
|  | Session 1 × Phase 2 | -2.29 | 4.56 | -0.50 | 0.616 |
|  | Session 1 × Phase 3 | -29.47 | 4.56 | -6.46 | **<0.001** |
|  | Session 1 × Phase 4 | 2.51 | 4.56 | 0.55 | 0.583 |
|  | Session 2 × Phase 2 | -4.38 | 4.76 | -0.92 | 0.359 |
|  | Session 2 × Phase 3 | -22.22 | 4.76 | -4.67 | **<0.001** |
|  | Session 2 × Phase 4 | 3.52 | 4.76 | 0.74 | 0.461 |
|  | Session 3 × Phase 2 | -3.03 | 4.76 | -0.74 | 0.526 |
|  | Session 3 × Phase 3 | -24.36 | 4.76 | -5.11 | **<0.001** |
|  | Session 3 × Phase 4 | 1.69 | 4.76 | 0.36 | 0.723 |

Table S3. Linear mixed-effects effects model (LMM) parameters for the main effects of *Session* and *Phase* on muscle activity in stationary load handling tasks, analyzed separately by dependent measures*.* All models included the *Phase* × *Session* interaction term; however, likelihood ratio tests (LRT) indicated that these interactions were not significant for any muscle activity; therefore, interpretations focused on the main effects. Statistically significant *p*-values are indicated in bold.

| **Dependent**  **Measures** | **Fixed Effect** | **Parameter**  **Estimate** | **Std. Error** | ***t* value** | ***p* value** |
| --- | --- | --- | --- | --- | --- |
| *Dominant Lumbar Erector Spinae (%MVIC)* |  |  |  |  |  |
| *5^th^ Percentile* | Intercept | 9.69 | 2.49 | 3.90 | **0.001** |
|  | Session 1 | 5.31 | 3.16 | 1.68 | 0.117 |
|  | Session 3 | 4.38 | 3.39 | 1.29 | 0.218 |
|  | Phase 2 | -0.70 | 0.90 | -0.78 | 0.434 |
|  | Phase 3 | -1.05 | 0.90 | -1.17 | 0.242 |
|  | Phase 4 | 0.85 | 0.90 | 0.94 | 0.346 |
| *50^th^ Percentile* | Intercept | 17.11 | 3.32 | 5.15 | **<0.001** |
|  | Session 1 | 5.65 | 4.29 | 1.32 | 0.211 |
|  | Session 3 | 4.37 | 4.60 | 0.95 | 0.360 |
|  | Phase 2 | -0.72 | 1.04 | -0.69 | 0.489 |
|  | Phase 3 | -0.78 | 1.04 | -0.75 | 0.454 |
|  | Phase 4 | 1.83 | 1.04 | 1.77 | 0.079 |
| *95^th^ Percentile* | Intercept | 28.72 | 4.50 | 6.38 | **<0.001** |
|  | Session 1 | 4.89 | 5.72 | 0.86 | 0.409 |
|  | Session 3 | 3.07 | 6.14 | 0.50 | 0.626 |
|  | Phase 2 | -0.25 | 1.36 | -0.18 | 0.855 |
|  | Phase 3 | -1.15 | 1.36 | -0.85 | 0.397 |
|  | Phase 4 | 1.43 | 1.36 | 1.05 | 0.293 |
| *Non-Dominant Lumbar Erector Spinae (%MVIC)* |  |  |  |  |  |
| *5^th^ Percentile* | Intercept | 9.90 | 2.35 | 4.21 | **<0.001** |
|  | Session 1 | 6.01 | 3.16 | 1.90 | 0.078 |
|  | Session 3 | 7.56 | 3.38 | 2.24 | **0.042** |
|  | Phase 2 | -2.11 | 1.06 | -1.99 | **0.048** |
|  | Phase 3 | -1.38 | 1.08 | -1.28 | 0.202 |
|  | Phase 4 | 1.77 | 1.08 | 1.63 | 0.104 |
| *50^th^ Percentile* | Intercept | 22.50 | 3.06 | 7.37 | **<0.001** |
|  | Session 1 | 0.71 | 3.85 | 0.19 | 0.856 |
|  | Session 3 | 3.38 | 4.12 | 0.82 | 0.427 |
|  | Phase 2 | -1.69 | 1.16 | -1.45 | 0.149 |
|  | Phase 3 | -1.42 | 1.18 | -1.12 | 0.231 |
|  | Phase 4 | 1.85 | 1.19 | 1.56 | 0.121 |
| *95^th^ Percentile* | Intercept | 37.33 | 3.62 | 10.31 | **<0.001** |
|  | Session 1 | -3.58 | 3.55 | -1.01 | 0.334 |
|  | Session 3 | -3.74 | 3.78 | -0.99 | 0.344 |
|  | Phase 2 | -0.39 | 1.68 | -0.23 | 0.816 |
|  | Phase 3 | -0.49 | 1.70 | -0.29 | 0.773 |
|  | Phase 4 | 3.87 | 1.71 | 2.27 | **0.025** |
| *Dominant Anterior Deltoid (%MVIC)* |  |  |  |  |  |
| *5^th^ Percentile* | Intercept | 6.13 | 0.89 | 6.86 | **<0.001** |
|  | Session 1 | -2.42 | 1.20 | -2.02 | 0.069 |
|  | Session 3 | -2.36 | 1.28 | -1.84 | 0.093 |
|  | Phase 2 | -1.44 | 0.73 | -1.98 | **0.049** |
|  | Phase 3 | -1.82 | 0.73 | -2.51 | **0.013** |
|  | Phase 4 | -2.57 | 0.73 | -3.51 | **<0.001** |
| *50^th^ Percentile* | Intercept | 23.03 | 2.62 | 8.78 | **<0.001** |
|  | Session 1 | -5.71 | 4.11 | -1.39 | 0.180 |
|  | Session 3 | -6.91 | 4.36 | -1.59 | 0.129 |
|  | Phase 2 | -0.64 | 1.69 | -0.38 | 0.707 |
|  | Phase 3 | -4.39 | 1.69 | -2.59 | **0.010** |
|  | Phase 4 | -8.12 | 1.70 | -4.77 | **<0.001** |
| *95^th^ Percentile* | Intercept | 64.01 | 6.91 | 9.27 | **<0.001** |
|  | Session 1 | -5.88 | 10.56 | -0.56 | 0.584 |
|  | Session 3 | -10.28 | 11.21 | -0.92 | 0.370 |
|  | Phase 2 | -6.45 | 5.08 | -1.27 | 0.206 |
|  | Phase 3 | -17.25 | 5.08 | -3.40 | **<0.001** |
|  | Phase 4 | -21.39 | 5.11 | -4.19 | **<0.001** |
| *Dominant Vastus Lateralis*  *Oblique (%MVIC)* |  |  |  |  |  |
| *5^th^ Percentile* | Intercept | 4.34 | 2.04 | 2.13 | **0.048** |
|  | Session 1 | 6.27 | 2.46 | 2.54 | **0.024** |
|  | Session 3 | 4.63 | 2.64 | 1.75 | 0.103 |
|  | Phase 2 | -0.57 | 0.87 | -0.65 | 0.515 |
|  | Phase 3 | 0.30 | 0.87 | 0.34 | 0.734 |
|  | Phase 4 | -1.21 | 0.87 | -1.39 | 0.165 |
| *50^th^ Percentile* | Intercept | 13.83 | 3.58 | 3.87 | **0.001** |
|  | Session 1 | 7.92 | 3.86 | 2.05 | 0.061 |
|  | Session 3 | 3.83 | 4.13 | 0.93 | 0.371 |
|  | Phase 2 | -2.40 | 1.70 | -1.41 | 0.160 |
|  | Phase 3 | 3.26 | 1.71 | 1.91 | 0.058 |
|  | Phase 4 | 0.62 | 1.70 | 0.36 | 0.717 |
| *95^th^ Percentile* | Intercept | 30.65 | 6.33 | 4.84 | **<0.001** |
|  | Session 1 | 9.80 | 6.20 | 1.58 | 0.142 |
|  | Session 3 | 0.79 | 6.63 | 0.12 | 0.907 |
|  | Phase 2 | -2.21 | 3.45 | -0.64 | 0.524 |
|  | Phase 3 | 5.85 | 3.47 | 1.69 | 0.093 |
|  | Phase 4 | 10.55 | 3.45 | 3.06 | **0.003** |
| *Non-Dominant Vastus Lateralis (%MVIC)* |  |  |  |  |  |
| *5^th^ Percentile* | Intercept | 4.48 | 2.61 | 1.72 | 0.102 |
|  | Session 1 | 7.44 | 3.42 | 2.17 | **0.048** |
|  | Session 3 | 7.39 | 3.67 | 2.02 | 0.063 |
|  | Phase 2 | -1.01 | 1.08 | -0.93 | 0.352 |
|  | Phase 3 | -0.05 | 1.08 | -0.04 | 0.966 |
|  | Phase 4 | -1.50 | 1.08 | -1.40 | 0.164 |
| *50^th^ Percentile* | Intercept | 14.78 | 4.50 | 3.29 | **0.004** |
|  | Session 1 | 13.32 | 4.50 | 2.96 | **0.012** |
|  | Session 3 | 9.25 | 4.82 | 1.92 | 0.079 |
|  | Phase 2 | -2.88 | 1.93 | -1.50 | 0.136 |
|  | Phase 3 | 0.66 | 1.93 | 0.34 | 0.734 |
|  | Phase 4 | -0.36 | 1.93 | -0.19 | 0.852 |
| *95^th^ Percentile* | Intercept | 32.81 | 7.40 | 4.43 | **<0.001** |
|  | Session 1 | 17.23 | 5.53 | 3.12 | **0.010** |
|  | Session 3 | 5.32 | 5.90 | 0.90 | 0.388 |
|  | Phase 2 | -0.10 | 3.46 | -0.03 | 0.976 |
|  | Phase 3 | 3.79 | 3.47 | 1.09 | 0.277 |
|  | Phase 4 | 7.46 | 3.46 | 2.16 | **0.032** |
| *Dominant*  *Biceps Femoris (%MVIC)* |  |  |  |  |  |
| *5^th^ Percentile* | Intercept | 3.49 | 0.69 | 5.06 | **<0.001** |
|  | Session 1 | 1.43 | 0.89 | 1.61 | 0.131 |
|  | Session 3 | 2.55 | 0.95 | 2.68 | **0.018** |
|  | Phase 2 | -0.46 | 0.45 | -1.02 | 0.308 |
|  | Phase 3 | -0.74 | 0.45 | -1.65 | 0.102 |
|  | Phase 4 | -0.18 | 0.45 | -0.39 | 0.694 |
| *50^th^ Percentile* | Intercept | 10.57 | 1.44 | 7.36 | **<0.001** |
|  | Session 1 | -0.84 | 1.49 | -0.56 | 0.583 |
|  | Session 3 | 1.19 | 1.60 | 0.75 | 0.470 |
|  | Phase 2 | 0.16 | 0.70 | 0.23 | 0.815 |
|  | Phase 3 | -1.51 | 0.70 | -2.16 | **0.032** |
|  | Phase 4 | 0.18 | 0.70 | 0.26 | 0.798 |
| *95^th^ Percentile* | Intercept | 22.32 | 2.61 | 8.54 | **<0.001** |
|  | Session 1 | -6.14 | 1.50 | -4.09 | **0.002** |
|  | Session 3 | -5.15 | 1.60 | -3.21 | **0.010** |
|  | Phase 2 | 0.97 | 0.93 | 1.04 | 0.300 |
|  | Phase 3 | -1.63 | 0.93 | -1.75 | 0.083 |
|  | Phase 4 | 0.01 | 0.93 | 0.02 | 0.988 |
| *Non-Dominant*  *Biceps Femoris (%MVIC)* |  |  |  |  |  |
| *5^th^ Percentile* | Intercept | 2.48 | 0.83 | 2.99 | **0.007** |
|  | Session 1 | 2.93 | 1.15 | 2.54 | **0.023** |
|  | Session 3 | 3.74 | 1.23 | 3.03 | **0.009** |
|  | Phase 2 | -0.73 | 0.46 | -1.60 | 0.111 |
|  | Phase 3 | -0.68 | 0.46 | -1.48 | 0.140 |
|  | Phase 4 | -0.22 | 0.46 | -0.48 | 0.634 |
| *50^th^ Percentile* | Intercept | 8.34 | 1.50 | 5.55 | **<0.001** |
|  | Session 1 | 2.70 | 1.57 | 1.72 | 0.110 |
|  | Session 3 | 5.52 | 1.68 | 3.29 | **0.006** |
|  | Phase 2 | 0.24 | 0.79 | 0.30 | 0.762 |
|  | Phase 3 | -1.89 | 0.79 | -2.39 | **0.018** |
|  | Phase 4 | -0.12 | 0.79 | -0.15 | 0.879 |
| *95^th^ Percentile* | Intercept | 20.00 | 2.83 | 7.06 | **<0.001** |
|  | Session 1 | -3.64 | 2.09 | -1.74 | 0.112 |
|  | Session 3 | 0.27 | 2.24 | 0.12 | 0.908 |
|  | Phase 2 | 2.20 | 1.13 | 1.95 | 0.053 |
|  | Phase 3 | -0.86 | 1.13 | -0.76 | 0.450 |
|  | Phase 4 | 0.33 | 1.13 | 0.29 | 0.771 |

**Table S4.** Linear mixed-effects model (LMM) parameters for the main effects of *Session* on RPE and NASA-TLX in stationary load handling tasks, analyzed separately by dependent measures*.* Statistically significant *p*-values are indicated in bold.

| **Dependent**  **Measures** | **Fixed Effect** | **Parameter**  **Estimate** | **Std. Error** | ***t* value** | ***p* value** |
| --- | --- | --- | --- | --- | --- |
| *Ratings of Perceived Exertion (RPE)* |  |  |  |  |  |
| *Arms* | Intercept | 5.72 | 3.06 | 1.87 | 0.096 |
|  | Session 1 | -2.00 | 1.31 | -1.53 | 0.157 |
|  | Session 2 | -1.35 | 1.33 | -1.02 | 0.332 |
|  | Session 3 | -0.92 | 1.31 | -0.70 | 0.499 |
| *Shoulders* | Intercept | 5.02 | 3.57 | 1.41 | 0.193 |
|  | Session 1 | -1.93 | 1.54 | -1.25 | 0.237 |
|  | Session 2 | -0.90 | 1.56 | -0.58 | 0.577 |
|  | Session 3 | -0.38 | 1.54 | -0.24 | 0.812 |
| *Upper back* | Intercept | 2.72 | 1.86 | 1.46 | 0.181 |
|  | Session 1 | -0.68 | 0.80 | -0.84 | 0.420 |
|  | Session 2 | -0.66 | 0.82 | -0.81 | 0.438 |
|  | Session 3 | -0.06 | 0.80 | -0.07 | 0.944 |
| *Lower back* | Intercept | 1.97 | 1.48 | 1.33 | 0.219 |
|  | Session 1 | -0.23 | 0.63 | -0.37 | 0.719 |
|  | Session 2 | -0.24 | 0.64 | -0.37 | 0.717 |
|  | Session 3 | 0.10 | 0.63 | 0.16 | 0.877 |
| *Hips* | Intercept | 2.86 | 1.61 | 1.78 | 0.111 |
|  | Session 1 | -0.74 | 0.72 | -1.03 | 0.324 |
|  | Session 2 | -0.62 | 0.74 | -0.84 | 0.418 |
|  | Session 3 | -0.38 | 0.72 | -0.52 | 0.613 |
| *Legs* | Intercept | 4.54 | 1.50 | 3.04 | **0.013** |
|  | Session 1 | -1.19 | 0.66 | -1.81 | 0.096 |
|  | Session 2 | -1.97 | 0.67 | -2.92 | **0.012** |
|  | Session 3 | -0.91 | 0.66 | -1.38 | 0.193 |
| *NASA-TLX* |  |  |  |  |  |
| *Mental* | Intercept | 7.69 | 2.56 | 3.00 | **0.013** |
|  | Session 1 | -3.75 | 1.21 | -3.10 | **0.007** |
|  | Session 2 | -1.61 | 1.26 | -1.28 | 0.220 |
|  | Session 3 | -0.58 | 1.21 | -0.48 | 0.637 |
| *Physical* | Intercept | 7.31 | 3.40 | 2.15 | 0.062 |
|  | Session 1 | -3.08 | 1.43 | -2.15 | 0.058 |
|  | Session 2 | -1.73 | 1.45 | -1.19 | 0.260 |
|  | Session 3 | -0.75 | 1.43 | -0.52 | 0.613 |
| *Temporal* | Intercept | 3.01 | 2.29 | 1.31 | 0.220 |
|  | Session 1 | -1.42 | 1.04 | -1.37 | 0.195 |
|  | Session 2 | -0.43 | 1.06 | -0.40 | 0.693 |
|  | Session 3 | 0.08 | 1.04 | 0.08 | 0.937 |
| *Performance* | Intercept | 4.02 | 1.92 | 2.10 | 0.058 |
|  | Session 1 | -2.00 | 0.99 | -2.03 | 0.058 |
|  | Session 2 | -0.19 | 1.04 | -0.18 | 0.858 |
|  | Session 3 | 0.17 | 0.99 | 0.17 | 0.868 |
| *Effort* | Intercept | 8.50 | 2.98 | 2.85 | **0.019** |
|  | Session 1 | -4.42 | 1.29 | -3.42 | **0.006** |
|  | Session 2 | -1.42 | 1.32 | -1.08 | 0.304 |
|  | Session 3 | -0.92 | 1.29 | -0.71 | 0.493 |
| *Frustration* | Intercept | 7.26 | 2.25 | 3.23 | **0.008** |
|  | Session 1 | -4.00 | 1.03 | -3.88 | **0.002** |
|  | Session 2 | -1.76 | 1.06 | -1.66 | 0.118 |
|  | Session 3 | -1.00 | 1.03 | -0.97 | 0.349 |
